# Sustaining employee wellbeing: a mediation analysis of resilience as a pathway from sustainable HRM to happiness in healthcare and wellness settings

**DOI:** 10.3389/fpsyg.2026.1789646

**Published:** 2026-04-13

**Authors:** Eglė Staniškienė, Živilė Stankevičiūtė, Asta Daunorienė, Joana Ramanauskaitė

**Affiliations:** School of Economics and Business, Kaunas University of Technology, Kaunas, Lithuania

**Keywords:** employee resilience, happiness at work, healthcare sector, partial mediation, sustainable HRM, wellness sector

## Abstract

**Introduction:**

Employee wellbeing has emerged as a critical concern for contemporary organizations, particularly in healthcare and wellness sectors where human capital is essential for service delivery. Although these professionals frequently encounter high demands, long working hours, and emotionally demanding tasks, sustaining their wellbeing represents both an ethical priority and strategic necessity for organizational sustainability. While research suggests that sustainable human resource management (HRM) practices foster employee wellbeing and long-term organizational performance, critical questions remain about the mechanisms through these practices operate. Specifically, it remains unclear whether sustainable HRM influences wellbeing solely through indirect pathways (such as building employee capacities) or also through direct effects, and whether these mechanisms operate similarly across different institutional and workforce contexts.

**Methods:**

We used a quantitative research strategy to collect data from 631 employees working in healthcare and wellness institutions in Lithuania during 2025. The questionnaire was distributed via organizational channels and professional networks. We analyzed the data using partial least squares structural equation modeling (PLS-SEM) with SmartPLS 4.0 software, employing bootstrapping procedures.

**Results:**

The measurement model demonstrated strong reliability and validity. Hypothesis testing revealed that sustainable HRM practices significantly and positively related to employee resilience, and employee resilience significantly related to happiness at work. Sustainable HRM also demonstrated a strong direct effect on happiness at work. The indirect effect through employee resilience was statistically significant, confirming partial mediation. Together, sustainable HRM and employee resilience explained 39.1% of variance in happiness at work, while sustainable HRM accounted for 8.0% of variance in employee resilience.

**Discussion:**

Research findings contribute to theoretical understanding by demonstrating that sustainable HRM influences happiness at work through dual pathways: directly through immediate perceptions of organizational support, fairness, and care, and indirectly through development of employee resilience as an adaptive capacity. This partial mediation pattern aligns with Conservation of Resources Theory and Broaden-and-Build Theory, revealing that sustainable HRM creates value through both immediate attitudinal enhancement and long-term resource development. Practically, results indicate that sustainable HRM including decent work, work place democracy and sustainable career climate could be presented as strategic investments that have directly impact to happiness at work and employee resilience.

## 1 Introduction

Contemporary healthcare and wellness organizations face an unprecedented challenge to maintain employee wellbeing amid escalating demands, workforce shortages, and emotional intensity that characterize these sectors (Bhattacharyya et al., 2019). In the post COVID working environment, these pressures have been taken research attention on protecting healthcare and wellness organizations employees wellbeing as a core element of health system functioning (McNeil, 2026). In this context, employee resilience, the capacity to adapt positively to organizational, social, and work environment challenges while maintaining functioning, has emerged as critical for both individual wellbeing and organizational sustainability (Näswall et al., 2015; Qamar et al., 2024). Recent paper have also emphasizes healthcare employee resilience through socio-ecological perspective, dealing that adaptive capacity is shaped not only by individual resources but also by organizational system factors that enable wellbeing (Pundir et al., 2025).

In this context, ensuring employee wellbeing is no longer a secondary condition, but rather a critical part of organizations' daily activities, closely linked to service quality, productivity, and long-term sustainability (Cooper et al., 2019; Kowalski and Loretto, 2017). Empirical research shows that taking care of healthcare and wellness sector professionals' wellbeing is linked to the different situations, such as challenges where organizations deal with employee burnout, stress, absenteeism, and turnover, or positive situations where wellbeing is associated with better work performance, organizational commitment, and service user outcomes (Lu et al., 2023; Sorribes et al., 2021). Recent systematic research shows that that factors art different levels of the organization influence employee wellbeing and related organizational outcomes, supporting the argument that wellbeing is a core organizational issue (Pandey et al., 2025). Post COVID research shows how working conditions impact healthcare wellbeing and resilience, showing that these constructs continue to be relevant in current healthcare settings (Alharbi, 2025).

The research emphasizes that the ability of employees to maintain wellbeing in challenging conditions depends not only on the intensity of work, but also on their ability to adapt to these demands and cope with them over time (Bhattacharyya et al., 2019). In this regard, employee resilience is defined as the ability to adapt positively to different conditions, recover from challenges, and maintain working in the face of stress. It is an important construct for understanding employee wellbeing in high-stress work environments (Näswall et al., 2015, 2019). At the organizational level, research from the pandemic shows that resilience-focused actions, such as clear internal communication about risks, psychosocial support, and crisis preparation, are linked to better wellbeing among healthcare employee, highlighting the importance of organizational practices in supporting resilience over time (Kosycarz et al., 2025).

In parallel with the development of resilience research, human resource management (HRM) researchers emphasize the importance of approaches that lead organizations toward more sustainable and people-oriented management systems (Stahl et al., 2020). Sustainable HRM emphasizes the development of human resources, balancing economic, social and environmental goals in the long term (De Prins et al., 2020; Macke and Genari, 2019). Rather than seeking high productivity at the cost of employee health, sustainable HRM in organizations seeks to avoid the decrease and loss of human capital by promoting fairness, participation, and employee wellbeing (Aust et al., 2020; Qamar et al., 2024). From organizational perspective, such practices can act as organizational resources that support employee capabilities to adapt and enable them to respond resiliently to ongoing challenges and uncertainty (Cooper et al., 2019) and experience happiness at work.

Despite growing importance that sustainable HRM supports employee wellbeing, the scientific literature is still fragmented on the discussions which HRM practices translate into positive effects (Lu et al., 2023; Qamar et al., 2024). While some studies show a direct positive effect on employee wellbeing (Almarzooqi et al., 2019), others are concerned that HRM systems, if poorly designed or managed, can increase job demands, work pressure, or intensify stress, in turn impacting employee health (Chillakuri and Vanka, 2020, 2021). These results indicate that sustainable HRM does not automatically lead to positive outcomes and highlight the need for research explaining when and how HRM practices lead to positive employee wellbeing, rather than assuming that the impact remains the same in all situations (Podgorodnichenko et al., 2020).

In this regard, employee resilience is a critical element helping the organizations influence employee performance and wellbeing (Näswall et al., 2019). Resilience is understood not as a fixed individual trait, but as a dynamic capacity in both personal and contextual situations (Bhattacharyya et al., 2019). In healthcare, resilience has been shown to protect against stress and burnout while maintaining engagement and wellbeing when faced with high job demands (Lu et al., 2023). By strengthening the employee ability to adapt to ongoing challenges, resilience can enable organizations to transform supportive practices into long-term positive experiences at work (Cooper et al., 2019).

From resilience research on positive attitudes toward employees, it has moved to wider concepts such as job satisfaction and engagement, which are usually analyzed separately (Fisher, 2010). Happiness at work has been proposed as a higher-level concept that incorporates engagement, job satisfaction, and emotional commitment to the organization, reflecting positive work experience of employees (Salas-Vallina and Vidal, 2018; Salas-Vallina et al., 2018). Unlike other indicators, which focus primarily on stress or poor wellbeing, happiness at work reflects positive attitude and is conceptually aligned with resilience as an adaptive process (Bhattacharyya et al., 2019). Research evidence shows that happiness at work is associated with learning abilities, organizational connection, and service quality, especially in service-intensive sectors such as healthcare and wellness (Salas-Vallina et al., 2017, 2020). However, little is known about how organizational practices promote happiness at work through employee abilities (Qamar et al., 2024).

While previous research has tested the relationships between sustainable HRM and employee resilience (Lu et al., 2023) and between sustainable HRM and happiness at work (Kocollari et al., 2023) important theoretical and empirical gaps remain. Existing mediation studies have focused primarily on testing whatever sustainable HRM operates solely through mediation mechanisms or also includes direct effects on wellbeing. Understanding whether mediation is partial or complete has important theoretical implications conceptualizing how organization practices influence employee outcomes. To date, few studies have integrated sustainable HRM, employee resilience, and happiness at work into a single mediation model, and none have examined this relationship using the three-dimensional happiness at work construct in healthcare and wellness contexts (Qamar et al., 2024).

Previous studies have examined the links between sustainable HRM, resilience and various indicators of work wellbeing, but these relationships have often been investigated separately. This study builds on existing literature by combining these variables into a single mediation model. This allows us to gain a more comprehensive understanding of how organizational practices lead to employee wellbeing outcomes through underlying psychological mechanisms.

Moreover, most empirical evidence are from Western European or East Asian contexts, leaving Central and Eastern European EU member states under-examined. This is problematic because healthcare systems in these regions face workforce and institutional pressures, including chronic staff shortages, persistent outmigration to higher-paying Western European positions, lower compensation relative to Western Europe, and ongoing public-sector reforms (European Commission, 2025). Those challenges may condition how sustainable HRM practices function and how important resilience is for maintaining wellbeing.

Lithuanian healthcare context presents distinct characteristics shaping the relevance of sustainable HRM for employee wellbeing. Healthcare organizations face workforce challenges including emigration of qualified professionals to higher-wage EU countries, aging workforce demographics, and recruitment difficulties particularly in regional facilities. These pressures create contexts where employee wellbeing assume critical strategic importance, as workforce losses directly threaten service delivery capacity. In parallel, Lithuanian wellness sector organizations, including spa resorts, sanatoriums, and rehabilitation centers, represent significant employment while facing similar workforce pressures alongside seasonal demand fluctuations and competitive pressures from international wellness destinations. Within these contexts, sustainable HRM practices may serve as crucial organizational responses to workforce challenges, though their effectiveness mechanisms require empirical investigation.

To address these gaps, this study tests a partial mediation model examining both direct and indirect pathways through which sustainable HRM influences happiness at work in Lithuanian healthcare and wellness organizations. We contribute to theory by testing whether employee resilience partially or fully mediates the sustainable HRM-happiness relationship, thereby revealing the mechanisms linking organizational practices to employee wellbeing. We also contribute context-specific evidence by testing this model in a Central and Eastern European healthcare system characterized by workforce pressures distinct from previously studied Western contexts. Our research question is: How does sustainable HRM influence happiness at work in healthcare and wellness organizations, and does employee resilience partially or fully mediate this relationship?

The paper body is organized as follows: Section 2 presents the theoretical background by reviewing literature on sustainable HRM, employee resilience, and happiness at work, and develops the hypotheses. Section 3 describes the methodology, including the sample, procedure, and measurement instruments. Section 4 reports the results, including the assessment of common method variance, descriptive statistics, measurement model evaluation, structural model testing, and mediation analysis. Section 5 discusses the findings and outlines theoretical and practical implications, followed by the study limitations and directions for future research. Finally, Section 6 concludes the paper.

## 2 Theoretical background and hypothesis development

**Figure 1:**
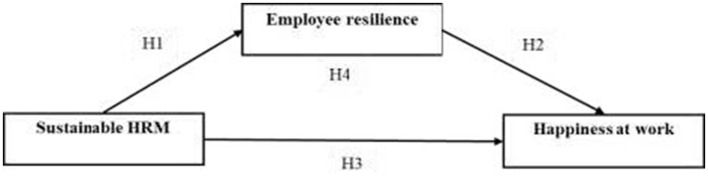
The research framework.

This study examines the relationships between sustainable HRM practices, employee resilience, and happiness at work in Lithuania healthcare and wellness sectors. Prior research links sustainable HRM to employee outcomes and highlights resilience as a potential driver, important gaps remain. Most mediation studies rely on cross-sectional evidence drawn mainly from Western European or East Asian settings, leaving Central and Eastern European EU member states, where healthcare systems face distinct workforce and capacity pressures, less examined. The relationship between resilience and the three-dimensional happiness at work construct has not yet been tested directly. Little is known about whether sustainable HRM operates similarly in healthcare and wellness environments shaped by rapid modernization, ongoing public-sector reform, and persistent resource constraints, which may condition the effectiveness of sustainable HRM practices. Addressing these gaps, this study tests a comprehensive mediation model in Lithuanian healthcare and wellness organizations, addressing the Lithuanian context to add context-sensitive evidence to sustainable HRM theory. The following sections develop the theoretical framework and hypotheses (Figure 1) by integrating general theory with considerations specific to Lithuania's contemporary healthcare and wellness landscape.

### 2.1 Sustainable HRM as organizational pathway to resilience

Sustainable HRM refers to HR policies and practices designed to support long-term organizational viability while taking care of employability and wellbeing of employees (De Prins et al., 2020). Unlike traditional HRM approaches that focus on short-term performance enhancement, sustainable HRM emphasizes employees as valuable resources requiring continuous investment. Core practices include fairness, participation, continuous development, and health protection that align HRM with broader sustainability objectives (Aust et al., 2020).

However, the sustainable HRM construct faces important conceptual and empirical challenges. Definitions vary considerably across studies (Souza et al., 2024). The next challenge is related to measurement inconsistency. Liang and Li (2024) emphasize that HRM studies still operationalize sustainable HRM through different practices and scales that limits comparability and theoretical integration. The relationship between sustainable HRM and employee outcomes varies by the context and conditions that differ across countries and cultures, so outcomes are not consistent across settings (Wojtczuk-Turek et al., 2025).

While several studies report positive positive connections between sustainable HRM practices and employee wellbeing outcomes including job satisfaction, work engagement, and organizational commitment Lu et al., (2023) important inconsistencies exists. The research results of (Lu et al. 2023) show, through mediation analysis, that sustainable HRM practices positively affect employee resilience, which leads to higher work engagement and improved performance. However the research findings were based on the manufacturing contexts, raising questions about connections to service sectors and different cultural settings. Moreover, literature reveals tensions and inconsistencies, which should be considered. While wellbeing-oriented HRM practices tend to enhance employee outcomes, performance-oriented HR systems may intensify job demands, potentially overlook wellbeing, particularly in organization with limited human resources (Bai, 2025). Chillakuri and Vanka (2020) showed that intensive work demands could harm employee health unless the organization supported its employees. These findings suggest that sustainable HRM effects are not universal but depend significantly on sectoral context, and resource availability and other aspects.

Cross-cultural studies across European healthcare systems (Sorribes et al., 2021) suggest that sustainable HRM practices are associated with lower turnover intentions, reduced burnout, and higher levels of employee wellbeing, although these relationships vary across national contexts, which is consistent with the Job Demands-Resources model explaining how job demands and resources shape burnout (Demerouti et al., 2001). This variation suggests that institutional, cultural, and sectoral factors moderate sustainable HRM effectiveness, a recognition that challenges simple transferability assumptions and underscores the need for contextualized research.

Lithuanian healthcare and wellness sectors face sustained workforce and resource pressures driven by ongoing reforms, rising service demand, and persistent labor shortages. Evidence points to comparatively lower pay than in Western Europe, staff outmigration, and intensified workloads, which together increase job demands while constraining organizational resources (European Commission, 2025). Although the wellness sector is growing and gaining international visibility, it often operates with tighter financial and human resource capacity than mature Western markets. In this context, organizational support becomes especially important, therefore, sustainable HRM practices may have stronger effects on employee resilience and happiness at work than in more resource-rich settings, implying that relationship strength is likely context dependent.

The theoretical connection between sustainable HRM and employee resilience can be explained through different frameworks. Conservation of Resources Theory suggests that sustainable HRM practices function as organizational resources that help employees build personal resource reserves, including resilience (Hobfoll et al., 2018). However, the Conservation of Resources Theory has been criticized for paying limited attention to how resources are actively used, a limitation that becomes particularly relevant when examining resilience as a dynamic process of adaptation rather than passive resource possession (Liao et al., 2022). This distinction becomes important for resilience, which is linked with active adaptation and recovery, rather than simply having more resources available. Accordingly, resilience is better understood as a dynamic process of positive adaptation, not a passive state (Robinson et al., 2024). When organizations provide fair conditions, development opportunities, and health protection, employees gain resources that enhance their adaptive capacity. The Ability-Motivation-Opportunity Framework suggest that sustainable HRM can build employee ability to cope with challenges, motivate adaptive responses, and provide opportunities to practice resilient behaviors (Mowbray et al., 2021). Together these theoretical insights support the importance of examining resilience as an active mediating process rather than treating resilience only as a resource end state.

Empirical evidence supports the role of sustainable HRM in fostering resilience, particularly in demanding contexts. Cooper et al. (2019) found that wellbeing-oriented HRM practices enhanced employee resilience, which subsequently improved performance despite high job demands. In healthcare and wellness settings, sustainable HRM practices have been identified as essential for building the resilience of employees who should be capable of maintaining service quality while protecting employee wellbeing (Bhattacharyya et al., 2019; Sorribes et al., 2021). However, most existing studies examine contexts with different institutional arrangements, workforce conditions, and cultural expectations.

The resilience as a building function of sustainable HRM is relevant in healthcare and wellness contexts, where employees regularly encounter emotionally challenging situations, ethical dilemmas, and limited resources (McCann et al., 2013). Sustainable HRM practices demonstrate organizational commitment to employee welfare, which enhances the perceived organizational support as a critical factor in coping with stress and resilience development (Hameed et al., 2021). By providing stable employment relationships, fair compensation, development opportunities, and participative decision-making, sustainable HRM creates conditions enabling employees to develop the resources for effective coping with challenges.

From a resilience perspective, sustainable HRM practices influence employee outcomes indirectly by shaping adaptive capacities, while also allowing for direct attitudinal effects on wellbeing (Cooper et al., 2019; Näswall et al., 2019). Sustainable HRM may therefore be described as an organizational resource management strategy that systematically pays attention to employee resilience, particularly in demanding work contexts. This resource-building attitude justifies sustainable HRM as a strategic approach generating benefits for both organizations and employees (Aust et al., 2020). Based on this theoretical reasoning and empirical evidence, the first hypothesis is formulated:

**H1:** Sustainable HRM practices are positively related to employee resilience.

### 2.2 Employee resilience connection to happiness at work

Happiness at work is a higher-order construct reflecting positive emotional experiences of employees about their work, job conditions, and organization (Salas-Vallina and Vidal, 2018; Salas-Vallina et al., 2018). Rather than analyzing engagement, job satisfaction, or affective commitment as isolated outcomes, happiness at work integrates these dimensions into a unified framework that captures employee positivity at work (Fisher, 2010). Within this framework, employee engagement reflects enthusiasm, energy, and performance in work tasks, job satisfaction covers evaluative judgments regarding job characteristics such as pay, advancement opportunities, and work conditions, and affective organizational commitment with considerations specific to Lithuania's contemporary represents emotional connection and sense of belonging to the organization (Salas-Vallina and Vidal, 2018). This integrative approach demonstrates the compatibility principle, suggesting that broad measures predict behavioral results better than narrow constructs (Harrison et al., 2006).

However, the happiness at work construct requires critical examination. First, cultural variations in what constitutes workplace happiness may limit universal applicability, as recent studies highlight cross-cultural differences in how happiness and wellbeing at work are defined and experienced (Kun et al., 2025). Second, the hierarchical factor structure linking engagement, satisfaction, and commitment requires further empirical validation across different countries and sectors, as the happiness at work may vary by the context.

The relationship between resilience and happiness at work can be understood through several theoretical perspectives. First, according to the Conservation of Resources Theory, resilience functions as a personal resource, seeing that resilient individuals are better positioned to acquire and maintain other resources, such as self-efficacy and optimism, which collectively enhance wellbeing and happiness (Hobfoll et al., 2018). Second, Broaden-and-Build Theory suggests that resilient individuals' capacity to maintain positive emotions during difficult situations helps build personal resources that foster happiness over time (Fredrickson, 2001; Cann and Kuiper, 2014). Third, resilience supports more effective emotional regulation, enabling individuals to keep positive attitudes, which are an important component of happiness at work (Smith and Hollinger-Smith, 2015).

Empirical results show the linkage between resilience and happiness. Bhattacharyya et al. (2019) demonstrated that resilience mediated the relationship between adaptive workplace humor and wellbeing among 354 healthcare professionals, suggesting that resilience served as a pathway to positive outcomes. Similarly, studies examining resilience and stress show that higher resilience levels correlate with reduced negative affect and enhanced positive emotions at work (Singh et al., 2019). In the healthcare sector, research during the COVID-19 pandemic revealed that resilience was associated with maintained wellbeing and job satisfaction despite stressors (Brunetto et al., 2021).

Furthermore, Lu et al. (2023) provided evidence that employee resilience mediated the relationship between sustainable HRM practices and employee outcomes, demonstrating that resilience served as an intermediate mechanism linking organizational resources to positive outcomes. The research of Liu et al. (2013) research confirmed that resilience stimulated positive work attitudes and behaviors among healthcare professionals, promoting wellbeing. These findings show the importance of testing the resilience mediating role empirically rather than assuming direct relationships between organizational practices and happiness.

Historical and ongoing societal and institutional shifts in Lithuania have made “resilience” and adaptive capacity particularly salient in both public and organizational discourse, suggesting a context where employees are accustomed to navigating uncertainty and change (Struberga et al., 2024). In the healthcare and wellness sectors, this matters because current evidence points to substantial job demands and workforce pressures, such as stressors affecting healthcare staff, projected shortages, and persistent attrition risks, which increase the importance of resilience for maintaining wellbeing (Kavaliauskas et al., 2024). Under such demanding conditions, resilience is likely to be a more consequential pathway for sustaining positive work-related wellbeing (including happiness at work) than in settings with stronger resources and lower uncertainty.

The connection between resilience and the three dimensions of happiness at work is a new research direction. Resilient employees are more likely to maintain high engagement because their adaptive capacities enable them to keep energy and enthusiasm despite challenges (Bakker and Demerouti, 2017). They experience greater job satisfaction as resilience allows them to interpret job characteristics more positively and cope effectively with challenges (Kuiper, 2012). Additionally, resilient employees maintain stronger affective organizational commitment because their ability to navigate organizational changes and challenges preserves the emotional bonds with the organization (Salas-Vallina et al., 2020). This proves that resilience is an important determinant of happiness at work. Based on this theoretical reasoning and empirical evidence, the second hypothesis is proposed:

**H2:** Employee resilience is positively related to happiness at work.

### 2.3 Direct impact of sustainable HRM on happiness at work

Sustainable HRM refers to HR practices balancing organizational performance with long-term employee wellbeing and social responsibilities (Ehnert et al., 2016; Kramar, 2014). Unlike efficiency-oriented HRM, sustainable HRM integrates employee health, dignity, fairness, employability, and work-life balance into HR policies, positioning wellbeing as a core objective.

Job Demands-Resources Theory explains the direct effect of sustainable HRM on happiness at work. Job resources such as autonomy, social support, development opportunities, and fair HR systems stimulate motivation and foster positive states (Bakker and Demerouti, 2017). Sustainable HRM practices including participative decision-making, supportive leadership, continuous training, fair rewards, and employment security function as stable organizational resources directly enhancing work engagement, job satisfaction, and affective commitment, the core dimensions of happiness at work (Salas-Vallina and Vidal, 2018). Importantly, job resources exert direct motivational effects, promoting positive emotions even when mediating factors are absent.

Social Exchange Theory provides a complementary explanation. Sustainable HRM signals that organizations value employees as human beings with long-term needs (Rhoades and Eisenberger, 2002). When employees perceive organizational care, they reciprocate with positive attitudes, satisfaction, emotional attachment, and enthusiasm directly aligned with the happiness at work conceptualization (Salas-Vallina et al., 2017).

Empirical research supports this direct linkage. Studies in healthcare show that sustainable HRM practices associate with higher job satisfaction, engagement, and affective commitment even when mediating variables are not modeled (De Prins et al., 2020; Peccei and Van De Voorde, 2019). Systematic reviews consistently conclude that sustainable HRM predicts employee wellbeing outcomes across contexts (Qamar et al., 2024), indicating the intrinsic capacity of sustainable HRM to generate positive work experiences. Moreover, happiness at work reflects not only individual states but also the way work is designed, managed, and valued organizationally (Fisher, 2010). Sustainable HRM directly addresses these conditions by promoting ethical management, supportive climates, learning opportunities, and fairness conditions shown to enhance employees' positive evaluations of their job and organization (Salas-Vallina et al., 2017; Warr, 2007).

In contemporary Lithuania, participative sustainable HRM can demonstrate respect and trust, and evidence shows HRM practices shape employees' psychosocial experience (Savanevičiene et al., 2025). However, voice effects depend on credibility, if participation is only symbolic, it may weaken attitudes rather than improve them (Nyfoudi et al., 2024). This is especially relevant in healthcare, where hierarchies, regulation, and workforce strain can constrain voice and make supportive HRM more consequential for wellbeing (Hague et al., 2025; Vanckavičiene et al., 2024). Because Lithuanian organizations vary in their level of HRM modernization, direct effects are also likely to differ across organizational types.

Accordingly, the following hypothesis is proposed:

**H3:** Sustainable HRM has a positive direct impact on happiness at work.

### 2.4 Mediating role of employee resilience

Integrating sustainable HRM and happiness at work perspectives, this study proposes that happiness at work can be understood as the outcome of both supportive organizational practices and employee capacity to adapt to demanding work environments. This position aligns with the Mediation Theory, which suggests that organizational practices influence employees through intermediate practices as well as through direct effects on employee attitudes (Preacher and Hayes, 2008).

Specifically, sustainable HRM practices provide organizational resources such as fairness, development opportunities, participative decision-making, and health-oriented support, that could demonstrate organizational care and long-term orientation to employee wellbeing (De Prins et al., 2020; Bai, 2025). However, employee ability to transform these resources into positive work attitudes depends on their resilience (Cooper et al., 2019; Lu et al., 2023).

The theoretical background connected to H1 and H2 development creates a chain where sustainable HRM practices lead to resilience in turn leading to happiness at work. Similarly, the Ability-Motivation-Opportunity Framework suggests that sustainable HRM builds the employee abilities and provides opportunities to develop resilience, which then enables employees to maintain the motivation and energy necessary for happiness at work (Mowbray et al., 2021).

The mediation analysis follows a sequential process. First, sustainable HRM practices build resilience by providing resources that enhance the employee capacities. Fair treatment reduces threat, provides development opportunities and increases self-efficacy; health protection maintains energy reserves (Näswall et al., 2019). Second, once developed, resilience enables employees to maintain positive attitudes toward their work, evaluate job conditions, and sustain emotional connections with their organization. Those are three components of happiness at work identified by Salas-Vallina and Vidal (2018).

The mediation pathway is supported by different research results. Lu et al. (2023) invoked serial mediation analysis to demonstrate that sustainable HRM practices enhanced employee resilience, which subsequently increased work engagement, one of the dimensions of happiness at work. Cooper et al. (2019) found that wellbeing-oriented HRM practices improved employee outcomes through their positive effects on resilience, suggesting an indirect pathway. In healthcare settings, Bhattacharyya et al. (2019) showed that resilience mediated the relationship between supportive workplace resources and wellbeing among healthcare professionals, providing evidence for resilience as a factor through which organizational conditions translated into positive employee states.

Although prior studies support resilience as a mediating process, important limitations remain. Scientific evidence is based on cross-sectional, self-report designs, which limits causal inference and raises concerns about common method bias (Nyfoudi et al., 2024). The existing research tends to focus on isolated antecedents, such as leadership behaviors or single HR practices, rather than examining resilience within comprehensive and coherent sustainable HRM systems, leaving unclear how system-level HR approaches shape employee resilience (Savanevičiene et al., 2025). Moreover, the empirical evidence is still concentrated in Western or relatively resource-rich healthcare and organizational contexts, despite growing recognition that institutional and workforce pressures shape how employee voice, support, and wellbeing processes operate (Hague et al., 2025). This is particularly salient given the substantial workforce strain and resource constraints documented in Lithuanian healthcare and related sectors (Vanckavičiene et al., 2024; European Parliament, 2025). Finally, no studies to date have tested resilience as a mediator between sustainable HRM and the integrated, three-dimensional happiness at work construct, instead, prior research has examined narrower outcomes in isolation. Together, these gaps suggest that while the theoretical case for resilience as a mediator is strong, systematic empirical testing within contemporary, high-demand contexts such as Lithuania remains necessary.

While some research suggests that organizational resources may substitute for personal resources under certain conditions (Hobfoll et al., 2018), the research evidence in healthcare and wellness sectors supports resilience as a complementary pathway linking HRM practices to positive employee existence (Lu et al., 2023; Sorribes et al., 2021). In high-demand healthcare contexts, where employees face constant stress and emotional challenges, both organizational support and personal capacities are necessary for sustaining happiness at work (Brunetto et al., 2021).

The integration of Hypotheses 1 and 2 provides the logical foundation for the mediation hypothesis. If sustainable HRM practices enhance employee resilience (H1) and if resilience promotes happiness at work (H2), then resilience should logically serve as a mechanism through which sustainable HRM practices influence happiness at work. This sequential relationship of sustainable HRM → resilience → happiness at work represents the core theoretical contribution of this study, explaining not only whether but how sustainable HRM practices enhance employee happiness in demanding healthcare and wellness contexts. Based on theoretical reasoning, empirical evidence, and integration of the previous hypotheses, the fourth hypothesis is formulated:

**H4:** Employee resilience mediates the relationship between sustainable HRM practices and happiness at work.

## 3 Methodology

### 3.1 Participants and procedure

The study was conducted in Lithuania and focused on employees working in the healthcare and wellness sectors. The chosen sectors were characterized as service-intensive sectors with inherent high emotional demands, workforce shortages, and increasing performance pressures. Lithuania provides a distinctive socio-economic and institutional setting, combining a healthcare system with a rapidly growing wellness and health tourism sector. This context of the country offered a meaningful opportunity to examine the ways sustainable HRM practices could enhance resilience and happiness in healthcare and wellness sectors.

Lithuania healthcare system is funded through mandatory health insurance contributions and general taxation. In 2021, national healthcare expenditure totalled 4.4 billion, representing 7.8% of GDP, and the sector employed over 50,000 individuals, around 39,000 of whom were clinical professionals (OECD, 2022; National Health Insurance Fund, (NHIF), 2022). Alongside hospitals and clinics, Lithuania's healthcare ecosystem comprises internationally recognized wellness institutions, including spas, sanatoriums, and rehabilitation centers. The country's wellness sector has gained global recognition, attracting nearly 290,000 international visitors in 2024, representing a 16% increase from the previous year (Lithuania State Department of Tourism, 2025). The sector was awarded the ITB Health Tourism Award 2025, highlighting its quality and infrastructure standards (ITB Berlin, 2025). The coexistence of healthcare institutions and wellness organizations provides a robust empirical context for investigating sustainable HRM and its impact on employee wellbeing.

The research data was collected in early 2025 using a cross-sectional survey design from employees working in two main sectors: healthcare institutions (e.g. hospitals, family clinics and medical centers) and wellness institutions (e.g. spas, sanatoria and rehabilitation centers). To ensure relevance to the study's focus on sustainability, inclusion criteria required the participants to be employed in organizations that demonstrated a public commitment to sustainability, such as through sustainability reports, formal certifications, or explicitly stated organizational values.

Before data collection, organizational representatives and professional networks were contacted to explain the study purpose and to obtain support for distributing the questionnaire. Participation was voluntary, and respondents were informed about the confidentiality and anonymity of their responses. The questionnaire was administered online and disseminated through organizational communication channels and professional networks commonly used by healthcare and wellness employees. Data collection remained open for approximately 3 months. A total of 631 valid responses were collected, with at least 250 respondents per sector. This sample size exceeded common thresholds recommended for mediation analysis and provided sufficient statistical power to test the proposed hypotheses (Fritz and MacKinnon, 2007).

Table 1 presents demographic and occupational characteristics of the respondents. The majority of respondents (70%) were employed in the healthcare sector, while 30% worked in the wellness sector. Regarding organizational ownership, 61% of participants were employed in public sector organizations, with the remaining 39% working in private sector institutions. This distribution is consistent with the predominance of public healthcare provision in many healthcare systems.

**Table 1:** Respondents' profile.

Variables	Percentage (%)
Healthcare sector	70
Wellness sector	30
Private sector	39
Public sector	61
Gender	
Male	21
Female	79
Age	
Generation Z (date of birth 2001 and later)	9
Generation Y (date of birth 1982–2000)	39
Generation X (born in 1961–1981)	42
Baby boom generation (born in 1943–1960)	10
Time worked for the organizations (job tenure)	
Under 1 year	11
1–3 years	20
3–5 years	17
5–10 years	15
10 years and over	37
Working in the healthcare/wellness sector is your calling	60

The sample showed a gender imbalance, where 79% of the respondents were females were and 21%—males. This gender distribution is representative of the healthcare and wellness workforce, which is characterized by female predominance, particularly in nursing positions (Boniol et al., 2019). The age distribution showed that Generation X (born in 1961-1981) constituted the largest cohort at 42%, followed closely by Generation Y/Millennials (born in 1982-2000) at 39%. Baby Boomers (born in 1943–1960) represented 10% of the sample, while Generation Z (born in 2001 and later) comprised the smallest cohort at 9%.

Job tenure demonstrated that a great proportion of respondents (37%) reported a tenure of 10 years or more, indicating a stable core of experienced professionals. Conversely, 48% of respondents had been with their current organisation for less than 5 years, including 11% with less than 1 year of service, 20% with 1–3 years, and 17% with 3–5 years of tenure. Mid-career professionals with 5–10 years of work experience comprised 15% of the sample. This distribution may reflect both long-term staff commitment and recent workforce turnover. Sixty per cent of respondents identified occupational motivation as working in the healthcare/wellness sector was their calling, while 40% characterized it as “just a job”. This finding suggests that while the majority of workers demonstrate a sense of purpose and intrinsic motivation, the others approach their work from a more transactional perspective.

The sample profile reflects a predominantly female, experienced workforce distributed across Generations X and Y, employed primarily in public healthcare organizations. The workforce shows stability, reflected by long-tenured employees while most employees have a sense of vocational calling despite the challenges of healthcare and wellness work.

### 3.2 Measurements

The validated scales were measured using validated research scales published in leading journals and proved by high validity and reliability results. The selected scales have been applied in organizational and healthcare research and demonstrate strong psychometric properties and high levels of reliability and validity in empirical studies (DeVellis, 2017). Respondents were asked to indicate their agreement with each statement using a 7-point Likert-type scale, where 1 meant “do not agree absolutely” and 7—“absolutely agree”, with higher scores indicating higher levels of the respective construct. As the scales had been drawn up in the English language, the questionnaire was translated into Lithuanian using the back-translation procedure, which is recommended to ensure conceptual and linguistic relevance in research (Beaton et al., 2000). First, the items were translated from English into Lithuanian by a bilingual researcher. Later, an independent bilingual translator back-translated the items into English. The original and back-translated versions were then compared, and minor discrepancies were discussed and resolved to ensure equivalence in meaning.

#### 3.2.1 Sustainable HRM

Sustainable HRM was measured using the framework of De Prins et al. (2020). The items captured such dimensions as decent work, workplace democracy, and sustainable career climate. These dimensions reflect a holistic approach to HRM that seeks to balance organizational performance with the protection and development of employee resources over time. The scale emphasizes employee recognition of actual HR practices rather than formal policies, making it suitable for examining sustainable HRM in this research.

#### 3.2.2 Employee resilience

Employee resilience was assessed using the Employee Resilience Scale (Näswall et al., 2015), which focused on adaptability, learning from challenges and maintaining performance under stress. The scale focuses on behaviors such as collaborating effectively with others, learning from mistakes, responding constructively to feedback, seeking support when needed, and using change as an opportunity for growth. This behavioral and context-sensitive construct distinguishes employee resilience from personality-based resilience measures and aligns closely with the focus on organizational aspects of resilience, such as sustainable HRM practices. In this research, respondents rated the extent to which each statement reflected their typical behavior at work.

#### 3.2.3 Happiness at work

Happiness at work was measured using the Happiness at Work Scale (Salas-Vallina and Vidal, 2018), which covers engagement, job satisfaction and affective organizational commitment. The scale focuses on employees' enthusiasm and energy toward their work, their sense of purpose and satisfaction with job characteristics, and their emotional connection to the organisation. By integrating these dimensions, the scale provides a comprehensive assessment of positive work-related attitudes and reflects sustained positivity at work. In this research, respondents indicated their agreement with each item.

## 4 Results

### 4.1 Common method variance

The data analysis followed a two-stage approach recommended for PLS-SEM analysis Hair et al., (2021). First, common method variance was assessed to ensure data quality. Second, descriptive statistics and correlations were examined to provide preliminary evidence for the hypothesis relationships. Third, the measurement model was evaluated to confirm reliability and validity. Fourth, the structural model was tested to examine direct relationships. Finally, mediation analysis was conducted to test the indirect effect of sustainable HRM on happiness at work through employee resilience. All analyses were performed using SmartPLS 4.0 with bootstrapping procedures (5,000 resamples) to assess statistical significance.

This study used SPSS 29.0 software. Harman's one-factor test was performed to assess any potential overall bias in the measurement tools. As the study was based on self-reported perception of dimensions such as sustainable human resource management, employee resilience and happiness at work, the potential risk of method variance was considered. Procedural measures were implemented to reduce such bias. Respondents were assured of anonymity and confidentiality, participation was voluntary, and all instruments used in the study were validated. All items of all constructs were entered into an unrotated exploratory factor analysis. The results of Harman's one-factor test indicated that the cumulative variance explained by the first factor was 29.29%. As this did not exceed 40%, it can be concluded that the problem of common method bias in this study is not serious.

### 4.2 Descriptive statistics

As shown in Table 2, sustainable HRM is significantly and positively correlated with employee resilience (*r* = 0.133, *p* < 0.001), and employee resilience is positively related to happiness at work (*r* = 0.195, *p* < 0.001). Furthermore, sustainable HRM is significantly and positively correlated with happiness at work (*r* = 0.565, *p* < 0.01). These results provide preliminary empirical support for hypotheses 1 and 2 of this study.

**Table 2:** Means, standard deviations, and correlations between variables.

Variables	Mean	SD	1	2
1. Employee resilience	4.64	1.19	–	
2. Sustainable HRM	4.16	1.21	0.133^**^	–
3. Happiness at work	4.46	1.10	0.195^**^	0.565^**^

^**^p < 0.01.

### 4.3 Measurement model evaluation

The model was tested using partial least squares structural equation modeling (PLS-SEM) analysis with SmartPLS v. 4.0 software. The PLS-SEM statistical technique was selected for its suitability to address potential issues related to data normality and its flexibility with respect to distributional assumptions. Following the methodological guidance of (Hair et al. 2021), PLS-SEM is well suited for analyzing complex theoretical models, as it enables the simultaneous evaluation of measurement and structural components while accommodating mediating relationships among constructs. The normality of the data was assessed using skewness and kurtosis indicators. As all values fall within the ± 1 interval, it can be concluded that there are no significant deviations from the normal distribution. The data are therefore suitable for parametric analyzes, and the analysis followed the standard two-step procedure recommended for reflective measurement models. Firstly, the measurement model was assessed, including indicator reliability, internal consistency reliability, convergent validity, and discriminant validity. Secondly, the structural model was assessed, including collinearity diagnostics (VIF), coefficient of determination (*R*^2^), path coefficients, statistical significance via bootstrapping and mediation analysis. All results were estimated using non-parametric bootstrapping with 5,000 resamples.

The reliability of the indicator was assessed by examining the outer loadings of the measurement items. As shown in Table 3, most loadings exceeded the recommended threshold of 0.708, indicating satisfactory reliability. All loadings were statistically significant. Therefore, the indicators were considered appropriate measures of their respective constructs. Although several indicators reported loadings below 0.708, they were retained because the reliability and validity measures remained acceptable.

**Table 3:** Measurement model.

Construct	Indicator	Loading	*t-*value	*p-*value
Happiness at work	COMMITM1	0.780	37.806	<0.001
	COMMITM2	0.729	29.363	<0.001
	COMMITM3	0.682	23.346	<0.001
	ENGAGEMENT1	0.650	19.616	<0.001
	ENGAGEMENT2	0.638	17.198	<0.001
	ENGAGEMENT3	0.699	21.957	<0.001
	JOBS1	0.664	18.124	<0.001
	JOBS2	0.669	21.068	<0.001
	JOBS3	0.712	24.301	<0.001
Employee resilience	ER1	0.540	4.788	<0.001
	ER2	0.620	6.930	<0.001
	ER3	0.599	5.160	<0.001
	ER4	0.563	4.167	<0.001
	ER5	0.607	5.211	<0.001
	ER6	0.592	5.092	<0.001
	ER7	0.690	7.622	<0.001
	ER8	0.818	21.415	<0.001
	ER9	0.799	20.703	<0.001
Sustainable HRM	SUST_HRM_DW1	0.572	17.483	<0.001
	SUST_HRM_DW2	0.651	20.697	<0.001
	SUST_HRM_DW3	0.821	57.556	<0.001
	SUST_HRM_DW4	0.777	36.039	<0.001
	SUST_HRM_SCC1	0.638	19.460	<0.001
	SUST_HRM_SCC2	0.771	42.824	<0.001
	SUST_HRM_SCC3	0.749	36.985	<0.001
	SUST_HRM_WD1	0.770	37.860	<0.001
	SUST_HRM_WD2	0.595	14.180	<0.001
	SUST_HRM_WD3	0.814	53.072	<0.001
	SUST_HRM_WD4	0.807	55.939	<0.001

The results presented in Table 4 demonstrate that all three constructs meet the recommended reliability and validity thresholds. The Cronbach's alpha coefficients range from 0.864 to 0.910, indicating strong internal consistency. The composite reliability (rho_c) values are also high (0.869–0.925), thus confirming the hypothesis that the indicators consistently measure their respective latent variables. The Average Variance Extracted (AVE) values are deemed to be acceptable: sustainable HRM exceeds the 0.50 threshold (AVE = 0.532), while happiness at work (0.480) and employee resilience (0.428) are slightly below the ideal cutoff. Nevertheless, both constructs continue to demonstrate acceptable convergent validity, as evidenced by composite reliability values that exceed 0.70 and indicator loadings that surpass 0.50. In general, the indicators of reliability and validity demonstrate that the measurement model is both robust and suitable for structural analysis.

**Table 4:** Cronbach's Alpha, composite reliability and convergent validity of the constructs.

Constructs	Cronbach's alpha	Composite reliability (rho_a)	Composite reliability (rho_c)	Average variance extracted (AVE)
Happiness at work	0.864	0.866	0.892	0.480
Employee resilience	0.889	0.883	0.869	0.428
Sustainable HRM	0.910	0.918	0.925	0.532

As shown in Table 5, all Heterotrait–Monotrait (HTMT) ratios are below the recommended threshold of 0.85, indicating discriminant validity among the constructs. The HTMT values range from 0.227 to 0.624, showing that each construct is distinct and does not overlap excessively with others. The relationship between employee resilience and happiness at work (HTMT = 0.410) and the relationship between sustainable HRM and employee resilience (HTMT = 0.227) demonstrate low shared variance. The strongest association (Sustainable HRM–Happiness, HTMT = 0.624) remains below the conservative 0.85 limit. Overall, these results confirm that the constructs measure different phenomena and that the model satisfies the discriminant validity requirements necessary for PLS-SEM analysis.

**Table 5:** Discriminant validity of the constructs based on HTMT.

Constructs	Happiness at work	Employee resilience	Sustainable HRM
Happiness at work			
Employee resilience	0.410		
Sustainable HRM	0.624	0.227	

### 4.4 Inner (structural) model evaluation

Before the evaluation of the structural model, collinearity among predictor constructs was examined using the Variance Inflation Factor (VIF). The VIF values ranged from 1.00 to 1.72, which is substantially below the commonly accepted threshold of 5.00. The findings suggest that multicollinearity is not a concern in the model, and the relationships between variables can be interpreted without risk of inflated regression coefficients or distorted path estimates. Consequently, all constructs were retained for subsequent structural analysis.

The explanatory power of the structural model was assessed using the coefficient of determination (*R*^2^). As demonstrated in Table 6, sustainable HRM accounted for 8% of the variance in employee resilience (*R*^2^ = 0.080). Furthermore, it was demonstrated that sustainable HRM, in conjunction with employee resilience, accounted for 39.1% of the variance in happiness at work (*R*^2^ = 0.391). These values reflect small to moderate explanatory power, which is common and acceptable in social science research, particularly in wellbeing models where human behavior is influenced by multiple contextual and individual factors. The obtained R^2^ values indicate that the model demonstrates adequate predictive capability for this study.

**Table 6:** Coefficient of determination (*R*^2^).

Constructs	*R*-square	*R*-square adjusted
Happiness at work	0.391	0.390
Employee resilience	0.080	0.079

### 4.5 Hypothesis testing

**Figure 2:**
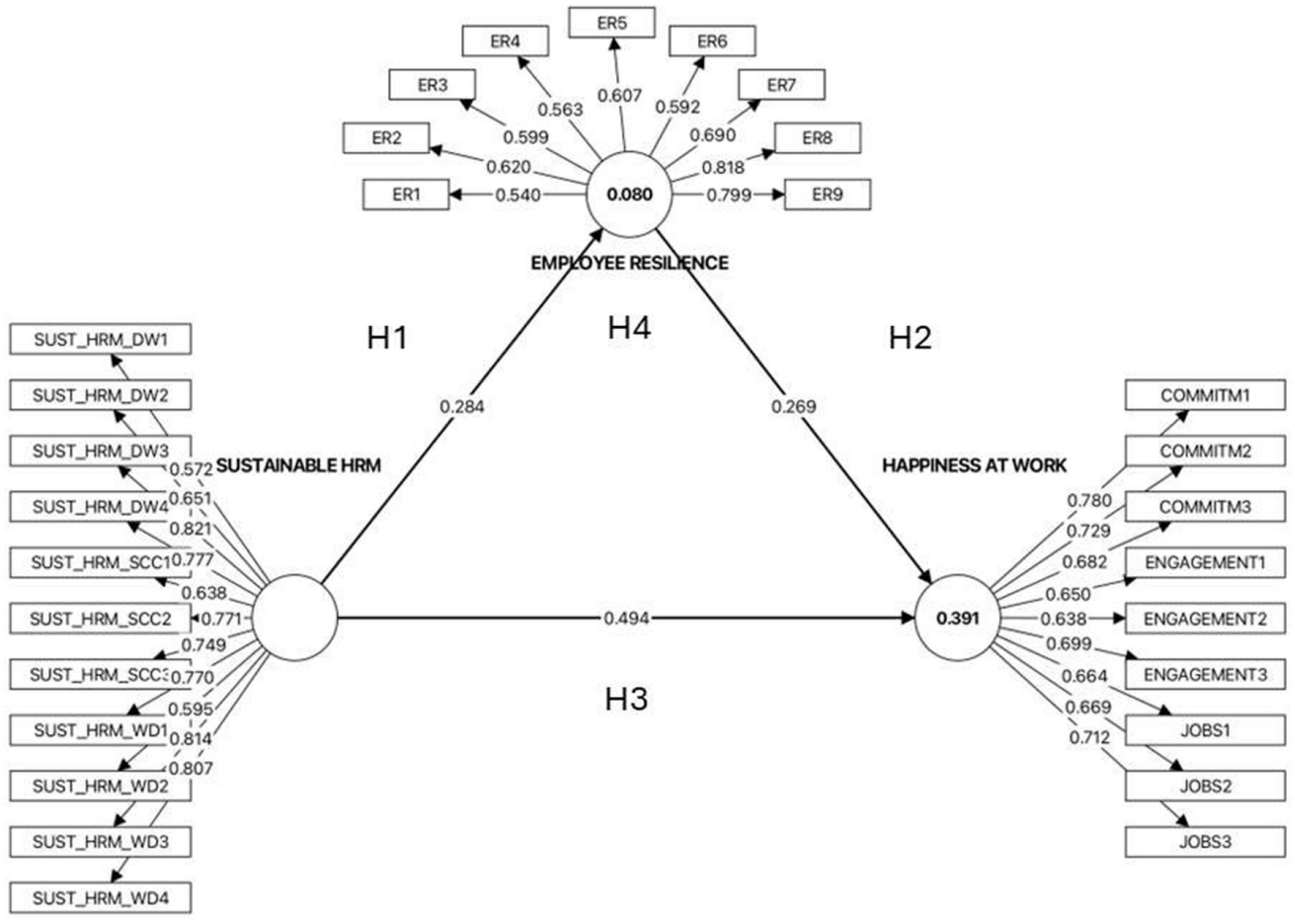
Measurement and structural model results (SmartPLS output).

As illustrated in Figure 2, the PLS analysis yielded several key findings, including the path coefficients and the variance explained by the structural model (in terms of *R*^2^ values).

The significance of the structural paths was assessed using the bootstrapping procedure with 5,000 resamples. The results demonstrated that all the hypothesized direct relationships in the model were positive and statistically significant. As demonstrated in Table 7, sustainable HRM was found to have a significant positive effect on employee resilience (β = 0.284, *t* = 6.708, *p* < 0.001), thus supporting H1. Furthermore, the findings of this study demonstrate that employee resilience exerts a significant positive influence on workplace happiness (β = 0.269, *t* = 7.519, *p* < 0.001), thereby substantiating Hypothesis 2. Next, sustainable HRM was found to have a strong, statistically significant direct effect on happiness at work (β = 0.494, *t* = 13.162, *p* < 0.001), thus supporting H3. Collectively, these findings imply that sustainable HRM exerts a substantial influence on the cultivation of employee resilience and workplace happiness, with resilience independently contributing to heightened levels of happiness at work.

**Table 7:** PLS-SEM inner model (direct effects).

Hypothesis	Relationship	Path coefficients	*t*-value	*p*-value	Decision
H1	Sustainable HRM → Employee resilience	0.284	6.708	*p* < 0.001	Supported
H2	Employee resilience → Happiness	0.269	7.519	*p* < 0.001	Supported
H3	Sustainable HRM → Happiness	0.494	13.162	*p* < 0.001	Supported

The mediating role of employee resilience (H4) was examined by testing the indirect effect using bootstrapping. As shown in Table 8, the indirect path ‘sustainable HRM → employee resilience → happiness at work' was statistically significant (indirect effect = 0.076, *t* = 5.324, *p* < 0.001), supporting H4. As both the direct (β = 0.494, *p* < 0.001) and indirect (β = 0.076, *p* < 0.001) effects remained significant, employee resilience partially mediates the relationship between sustainable HRM and happiness at work.

**Table 8:** Specific indirect effects (Mediation).

Hypothesis	Relationship	Path coefficients	*t*-value	*p*-value	Decision
H4	Sustainable HRM → Resilience → Happiness	0.076	5.324	*p* < 0.00	Supported partially

## 5 Discussion

### 5.1 General discussion of findings

This study examined how sustainable HRM practices contribute to foster employee resilience and happiness at work in Lithuanian healthcare and wellness sectors. Overall, the findings demonstrate that sustainable HRM practices enhance happiness at work through two pathways: a direct pathway reflecting immediate positive employee responses and an indirect pathway operating through the development of employee resilience. This dual-pathway structure suggests that organizational practices influence wellbeing not only by shaping employees day-to-day experiences but also by strengthening their longer-term adaptive capacity in demanding work environments.

The results are consistent with prior research demonstrating positive associations between sustainable HRM practices and employee resilience (supporting H1). This aligns with the Conservation of Resources Theory (Hobfoll et al., 2018). The theory shows that organizational resources facilitate personal resource development enabling individuals to better cope with stress. In the Lithuanian healthcare and wellness context, where employees are exposed to chronic stressors, emotional labor, and resource constraints, sustainable HRM practices, appear to function as critical organizational resources that enhance their adaptive capacity. This finding reinforces the multidimensional nature of resilience, shaped by organizational and individual factors (Näswall et al., 2019).

Employee resilience was also positively related to happiness at work (supporting H2). Resilient employees reported higher engagement, job satisfaction, and affective commitment, the three dimensions of happiness at work (Salas-Vallina and Vidal, 2018). This finding supports the Broaden-and-Build Theory (Fredrickson, 2001), suggesting that resilient individuals have the capacity to maintain positive emotions and build personal resources that foster happiness at work. In healthcare and wellness settings where employees encounter emotionally challenging situations and ethical dilemmas (Bhattacharyya et al., 2019), resilience enables keeping positive attitudes despite the demanding conditions.

In addition to the indirect pathway, sustainable HRM practices exerted a direct effect on happiness at work (supporting H3). This finding aligns with Job Demands-Resources Theory (Bakker and Demerouti, 2017) and Social Exchange Theory (Rhoades and Eisenberger, 2002), which suggest that organizational resources directly enhance motivational processes and positive work attitudes. In contexts such as Lithuanian healthcare and wellness sectors, where employees experience uncertainty, resource constraints, these immediate signals of organizational support may be particularly salient. When healthcare and wellness organizations implement sustainable HRM practices, including fair treatment, participative decision-making, development opportunities, and health protection, employees immediately perceive organizational support and care, which directly foster engagement, job satisfaction, and affective commitment.

The mediation analysis revealed that employee resilience partially mediated the sustainable HRM-happiness relationship (supporting H4). This partial mediation provides important insights. The indirect pathway shows sustainable HRM enhances happiness by building adaptive capacities, consistent with resource-building mechanisms (Hobfoll et al., 2018). The direct pathway operates through immediate perceptions of organizational support, fairness, and care, directly enhancing positive attitudes (Hameed et al., 2021). Together, sustainable HRM and resilience explain the variance of happiness at work, demonstrating predictive power in high-demand healthcare contexts facing workforce shortages, emotional demands, and performance pressures (OECD, 2022).

When interpreting the findings, it is important to note that the average variance extracted (AVE) values for employee resilience and happiness at work were marginally below the recommended threshold of 0.50. Although the composite reliability indicators met acceptable standards, the lower AVE values suggest that the constructs may not fully capture the underlying phenomena. Interpret relationships involving these variables cautiously. Nevertheless, the observed effects remain theoretically meaningful and consistent with prior research. Consequently, rather than providing definitive confirmation, the results should be understood as offering supportive evidence for proposed model.

### 5.2 Theoretical contributions

This research study makes several significant theoretical contributions to scientific research on sustainable HRM, employee resilience, and happiness at work by advancing understanding at a context specific, messo level, rather than attempting broad generalization.

Study contributes to sustainable HRM understanding by empirically testing a partial mediation model linking sustainable HRM practices to happiness at work through employee resilience. While previous research documented positive associations between sustainable HRM and wellbeing (Almarzooqi et al., 2019; Lu et al., 2023), the underlying mechanisms have remained underexplored the underlying mechanisms have remained underexplored. By demonstrating that sustainable HRM influences happiness through resilience addresses calls for analyzing intermediate processes (Podgorodnichenko et al., 2020; Qamar et al., 2024). The partial mediation reveals that sustainable HRM creates value through immediate impact of dual pathways and gradual adaptive capacity development.

The study enriches resilience research by testing sustainable HRM as an organizational antecedent in healthcare contexts. Previous research focused on individual factors or specific leadership styles (Bhattacharyya et al., 2019; Cooper et al., 2019). By analyzing sustainable HRM to cultivate resilience, this broadens the understanding of organizational conditions fostering adaptive capacity.

The study contributes to the happiness at work literature by identifying sustainable HRM as a key organizational driver of happiness at work both directly and indirectly via resilience. While happiness at work integrates engagement, job satisfaction, and affective commitment (Salas-Vallina et al., 2018), antecedent research remained limited (Qamar et al., 2024). The finding that sustainable HRM practices explain a substantial proportion of variance in happiness at work underscores the importance of organizational context and daily work experiences in fostering employee happiness in demanding sectors.

Consistent with recent discussions on theory development in context-specific settings (Homer and Lim, 2024), this study offers a small but meaningful theoretical extension rather than a universal theoretical reformulation. By situating the model within the Lithuanian healthcare and wellness sector, the findings demonstrate how sustainable HRM theory operates under conditions of high emotional demands, workforce shortages, and constrained resources. In doing so, the study highlights contextual elements that may challenge or nuance core assumptions of general HRM and wellbeing theories, thereby contributing a context-bound refinement rather than a generalisable claim.

The study contributes methodologically by applying PLS-SEM to a large healthcare and wellness sample (*N* = 631) using validated measurement scales (De Prins et al., 2020; Näswall et al., 2015; Salas-Vallina and Vidal, 2018). This provides a robust empirical foundation for future longitudinal and cross-cultural studies seeking to examine causal processes and contextual variation in sustainable HRM outcomes.

### 5.3 Practical implementations

The findings offer several actionable implications for healthcare and wellness organizations seeking to enhance employee resilience and happiness at work. Sustainable HRM practices should be viewed as strategic investments that generate both immediate and developmental returns. Practices such as fair treatment, participative decision-making, development opportunities, and health protection directly enhance employees' happiness at work, even in resource-constrained environments. This immediate positive impact underscores the value of prioritizing sustainable HRM initiatives despite operational pressures and competing demands.

The results also highlight the importance of recognizing employee resilience as a meaningful intermediate outcome of sustainable HRM. The significant indirect effect observed in this study indicates that sustainable HRM practices gradually strengthen employees adaptive capacities, which in turn contribute to higher levels of happiness at work. Organizations may therefore benefit from systematically assessing whether their HRM practices effectively foster resilience and from using validated instruments to monitor adaptive capacity over time. Such assessments can also help identify employee groups that may require additional support.

The study further suggests that organizations should implement sustainable HRM as a coherent system rather than as a set of isolated practices. Sustainable HRM encompasses multiple dimensions, including decent work conditions, workplace democracy, and a sustainable career climate. Evaluating existing HR practices against these dimensions can help organizations identify gaps, strengthen weaker areas, and ensure internal consistency across HR policies and practices.

Managerial implementation of sustainable HRM should explicitly account for both short-term employee wellbeing and long-term resilience development. Training programmes and managerial guidelines can emphasize how HR practices simultaneously enhance immediate happiness at work and build adaptive capacity over time. This dual perspective may support more informed decision-making regarding resource allocation and HR priorities, particularly in high-demand environments.

The findings are especially relevant for Lithuanian healthcare and wellness organizations facing persistent workforce shortages, high emotional demands, and increasing quality expectations. The evidence that sustainable HRM fosters both resilience and happiness at work despite these challenges demonstrates the potential of organizational practices to meaningfully support employee wellbeing even under strained conditions. Complementary initiatives, such as resilience training, peer support mechanisms, and mentoring programmes, may further enhance the effectiveness of sustainable HRM by addressing both organizational resources and individual capacity development.

### 5.4 Policy recommendations

Building on the practical implications, the findings also offer important policy relevant insights for both organizational decision-makers and public authorities seeking to improve employee wellbeing and workforce sustainability in healthcare and wellness sectors. Given the dual role of sustainable HRM in generating immediate wellbeing benefits and fostering long-term employee resilience, supportive policies at multiple levels can play a critical role in encouraging wider adoption of these practices.

At the organizational level, healthcare and wellness organizations are encouraged to formalize sustainable HRM principles within internal policies and strategic frameworks. Embedding fairness, participative decision-making, employee development, and health protection into organizational HR policies can help ensure that sustainable HRM practices are implemented consistently rather than on an *ad hoc* basis. Organizations may benefit from integrating employee wellbeing and resilience indicators into performance management and quality assurance systems, thereby signalling that employee sustainability is a strategic priority alongside service quality and financial performance. Such policies can strengthen workforce stability, reduce burnout and turnover, and ultimately support continuity and quality of care.

Organizational policies should also support managerial capability development. Providing managers with training and guidance on how sustainable HRM practices simultaneously enhance immediate employee happiness and longer-term resilience can facilitate more effective implementation. Clear internal policies that link sustainable HRM to leadership expectations may encourage managers to adopt practices that support employee wellbeing, even in high-pressure environments.

At the governmental level, public policy can play a crucial enabling role by creating conditions that support the adoption of sustainable HRM across the healthcare and wellness sectors. National healthcare workforce strategies could explicitly recognize employee wellbeing and resilience as policy objectives, encouraging organizations to move beyond short-term staffing solutions toward sustainable workforce management. Regulatory frameworks and accreditation standards may incorporate criteria related to decent work conditions, employee participation, and health protection, thereby incentivising organizations to implement sustainable HRM practices.

Financial and institutional support mechanisms may further encourage adoption. Governmental funding schemes, subsidies, or incentives linked to workforce development and employee wellbeing initiatives could reduce the perceived cost barriers associated with sustainable HRM implementation, particularly for smaller organizations. Public investment in training programmes focused on sustainable HRM and resilience development could also enhance organizational capacity and promote knowledge diffusion across the sector.

At a broader level, aligning sustainable HRM with national public health and labor policies may generate societal benefits. By fostering resilient and satisfied healthcare employees, such policies can contribute to reduced absenteeism, improved service quality, and greater system-level sustainability. In the Lithuanian context, where healthcare systems face persistent workforce shortages and increasing demands, policy support for sustainable HRM represents a proactive approach to strengthening both employee wellbeing and long-term healthcare system resilience.

## 6 Limitations and future research

This study focus on the Lithuanian healthcare sample limits generalisability of its findings to other national contexts, healthcare system types, and cultural settings. Lithuania's specific historical trajectory characterized by post-transitions reforms, EU integration, ongoing workforce emigration pressures, creates a unique institutional context that may shape how sustainable HRM practices operate and how employees respond to them. Consequently, mechanisms identified here might operate differently in healthcare systems with different development paths (e.g., Western European welfare states, US market-based systems), workforce conditions and cultural orientations toward participation, organizational authority, and work-life balance.

Future research should replicate the model across diverse national, cultural and organizational contexts to assess whether the dual-pathway structure and partial mediation reflect universal mechanisms or context-contingent patterns. Comparative studies Western European, Nordic, Southern European, and other post-transition countries would be particularly valuable for identifying which findings could be transferd broadly vs. which reflect Lithuanian specificities.

The substantial direct effect of sustainable HRM on happiness at work also suggests that additional explanatory mechanisms remain unexamined. Future studies should investigate alternative mediators, such as perceived organizational support, work meaningfulness, or human capital development, to provide a more comprehensive description of how sustainable HRM influences employee happiness.

Methodologically, the operationalisation of employee resilience and happiness at work constructs should be refined to improve their convergent validity. In addition, although Harman's single-factor test to assess common method bias, prior research indicates that this technique is limited and inconclusive. Because the data collection design did not include marker variables or the temporal, psychological or methodological separation of measures, common method variance cannot be fully ruled out. Accordingly, the reported relationships should be interpreted with caution, and future research should employ more robust procedural and statistical remedies to address this issue.

Finally, this study did not examine moderating effects and contextual factors are likely to influence how sustainable HRM fosters resilience and how resilience translates into happiness at work. Future research should test these boundary conditions to better understand variability in the proposed relationships.

## 7 Conclusions

This study examined the mechanisms through which sustainable HRM practices foster employee wellbeing in Lithuanian healthcare and wellness organizations, revealing critical insights into how organizational practices shape resilience and happiness at work in demanding professional contexts.

These findings provide theoretically meaningful and empirically supportive evidence for the proposed model, although measurement limitations warrant cautious interpretation. Sustainable HRM practices directly enhance happiness at work through immediate perceptions of organizational support, fairness, and care, an effect that proves substantial (β = 0.494) even in resource-constrained settings. Second, sustainable HRM builds employee resilience as an adaptive capacity, which subsequently contributes to happiness at work through an indirect pathway (β = 0.076). Together, these mechanisms explain 39.1% of variance in happiness at work, demonstrating that organizational practices meaningfully influence employee wellbeing even amid the chronic stressors, emotional demands, and workforce pressures characterizing Lithuanian healthcare and wellness sectors.

The theoretical contribution of this research lies in advancing beyond simple direct-effects models to reveal the complex, dual-pathway influence of sustainable HRM on wellbeing. By demonstrating partial mediation, this study addresses long-standing questions about how and when sustainable HRM practices translate into positive outcomes, responding scientific discussions (Podgorodnichenko et al., 2020; Qamar et al., 2024) for investigating intermediate processes rather than assuming uniform effects across contexts. The findings integrate Conservation of Resources Theory, Social Exchange Theory, and Broaden-and-Build Theory within a unified explanatory framework, showing that sustainable HRM creates value through both immediate enhancement and gradual resource development. Importantly, this research demonstrates that these theoretical mechanisms operate effectively in a post-transition Eastern European context, adding a Lithuanian healthcare and wellness sector piece to broader sustainable HRM theory while showing how universal mechanisms may be impact by the context-specific situations.

Methodologically, this study contributes by providing rigorous evidence from a substantial sample (*N* = 631) using validated instruments and advanced PLS-SEM analysis. While limitations exist, particularly the cross-sectional design preventing causal inference and marginal AVE values suggesting room for measurement refinement, the convergence of findings with theoretical predictions and prior research strengthens confidence in the results. The application of Western-developed scales in a Lithuanian context provides initial evidence of construct validity across cultural settings, enabling future comparative research.

For practitioners, this research offers evidence-based guidance with direct applicability. Sustainable HRM practices encompassing decent work conditions, workplace democracy, and sustainable career climate, represent strategic investments for meaningfull returns. Organizations implementing these practices can expect both immediate enhancements in employee engagement, satisfaction, and commitment, alongside longer-term development of resilient as adaptive capacities enabling sustained wellbeing. In Lithuanian healthcare and wellness organizations facing workforce shortages, high demands, and quality pressures, these findings demonstrate that systematic attention to sustainable HRM provides a pathway to employee wellbeing and organizational sustainability.

The policy implications extend across organizational and governmental levels. Organizations should develop sustainable HRM systems rather than isolated practices. Governments should incorporate sustainable HRM requirements into healthcare sector regulation, provide financial incentives supporting adoption, develop national workforce wellbeing standards. Such multi-level policy coordination could transform Lithuanian healthcare and wellness sectors toward sustainable HRM benefiting employees, organizations, and ultimately, the citizens relying on these essential services.

While these findings advance theoretical understanding and offer practical guidance, their generalizability requires careful consideration. The study examines Lithuanian healthcare and wellness organizations operating within specific institutional, economic, and cultural contexts that may not fully characterize healthcare systems elsewhere. The dual-pathway with substantial direct effects alongside resilience mediation may reflect Lithuanian healthcare situation, including resource constraints, workforce pressures dynamics, rather than representing universal sustainable HRM mechanisms. Practitioners and policymakers in other contexts should consider these findings as potentially relevant insights requiring adaptation to local realities rather than as universally applicable practices. Academic readers should view this research as contributing a Lithuanian piece to broader sustainable HRM theory, demonstrating how theoretical mechanisms operate in one specific context while remaining open to the possibility that emphasis patterns, effect sizes, or even pathway structures might differ in healthcare systems with different characteristics. By prioritizing sustainable HRM, treating employees as valuable long-term resources deserving of investment rather than expendable inputs, healthcare and wellness organizations can build resilient workforces capable of maintaining positive functioning and delivering high-quality care despite demanding environments.

## Data Availability

The raw data supporting the conclusions of this article will be made available by the authors, without undue reservation.
